# Central Retinal Vein Occlusion with Three-Retinal Quadrant Involvement: Another Focus on Optic Disc Head Vascular Anatomy Variations

**DOI:** 10.1155/2023/6648367

**Published:** 2023-10-31

**Authors:** Narges Hassanpoor, Vahid Abdolrahimi, Mohamad Reza Niyousha

**Affiliations:** ^1^Retina & Vitreous Service, Nikookari Eye Hospital, Tabriz University of Medical Sciences, Tabriz, Iran; ^2^Retina & Vitreous Service, Tehran University of Medical Sciences, Tehran, Iran

## Abstract

A 50-year-old male patient with sudden visual acuity loss in his right eye came to our clinic. Visual acuity at presentation was 1/10 in right eye and 10/10 in left. The patient was otherwise healthy Caucasian man without any history of previous systemic or ophthalmic disease. There was not any history of amblyopia and refractive error. Anterior segment findings were unremarkable. Three quadrants of retina were fully involved with central retinal vein occlusion (CRVO) features including retinal hemorrhages, retinal edema obscuring retinal details, and cotton wool spots while sparing inferior temporal quadrant. Inferior temporal quadrant sparing in this patient is due to a specific retinal vascular anatomical variation. In conclusion, in unusual presentations of retinal vascular branch obstructions, considering retinal vascular anatomy variations would help us to explain the clinical presentation more precisely in some cases.

## 1. Introduction

Retinal vascular occlusive diseases are one of the most common causes of visual loss. Among them, retinal vein occlusions are much more common than retinal artery occlusions [1–2]. As retinal vascular occlusive disease presentation is highly dependent to central retinal vein and artery anatomical variations, being familiar with these anatomical variations would help us with better diagnosis and management of the patients. Unusual presentations of retinal vascular occlusive diseases due to anatomical rare variations could be misdiagnosed as other retinal diseases [1–3].

## 2. Case Presentation

**Figure 1:**
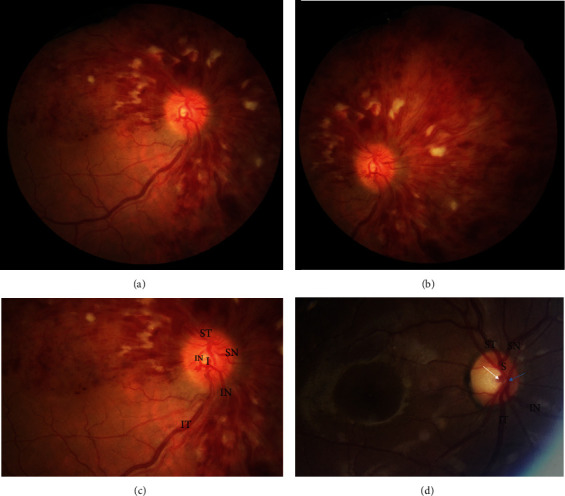
(a) The patient right eye fundus photo. Three quadrants of retina are fully involved with central retinal vein occlusion (CRVO) features including retinal hemorrhages, retinal edema obscuring retinal details, and cotton wool spots while sparing inferior temporal quadrant. (b) Nasal quadrant full involvement. (c) The patient optic nerve head vascular anatomy from a closer view. There is not a single venular trunk visible, but instead of that, there is a superior branch that gives three vascular branches for superior temporal (ST), superior nasal (SN), and inferior nasal (IN) veins. There is also an inferior retinal vein without any branch that drains inferior temporal quadrant that is intact in this case, however showing a slight engorgement. (d) A normal subject fundus photo for comparison. There is a single arterial end of the central retinal artery (blue arrow), and it is divided into superior and inferior branches; the superior one gives superior nasal and superior temporal arteries. The inferior branch gives inferior nasal and inferior temporal arteries. There is also a single venular trunk of central retinal vein (white arrow) divided into superior (S) and inferior (I) branches; the superior one gives superior nasal (SN) and superior temporal (ST) veins. The inferior branch gives inferior nasal (IN) and inferior temporal (IT) veins.

A 50-year-old male patient with sudden visual acuity loss in his right eye came to our clinic. Visual acuity at presentation was 1/10 in right eye and 10/10 in left. The patient was otherwise healthy Caucasian man without any history of previous systemic or ophthalmic disease. There were not any history of amblyopia and refractive error. Anterior segment findings were unremarkable. Intraocular pressure with Goldmann's applanation tonometer was 18 mm Hg in his both eyes. As evident in Figure 1(a), three quadrants of retina are fully involved with central retinal vein occlusion (CRVO) features including retinal hemorrhages, retinal edema obscuring retinal details, and cotton wool spots while sparing inferior temporal quadrant. Systemic workup, including HbA1c, complete blood count, homocysteine level, protein C, protein S, and factor 5 Leiden, and rheumatic disease workup were within normal limits. Blood pressure was 125/80.

Details of vascular supply in the optic disk (Figure 1) clear that inferior temporal retinal vein is not separated from main superior vein trunk. Actually, there are two main venular trunks at the optic nerve head, and the superior trunk is divided to three branches: superior temporal, superior nasal, and inferior nasal branches. The inferior main venular trunk drains blood from inferior temporal part of retina. The three branches of superior venular trunk supply the involved quadrants of patient retina proving to have common origin. Inferior trunk supplies inferotemporal quadrant that is not involved in this patient; this inferior trunk has no division. Inferior temporal quadrant sparing in this patient is due to retinal vascular anatomical variation.

Unfortunately, to the best of our review, there is no published data regarding central retinal vein anatomical variations and their prevalence; however, in our experience, the most common variation for retinal artery and vein could be like the one shown in Figure 1(d). As it is shown, there is a single arterial end of the central retinal artery (blue arrow), and it is divided into superior and inferior branches; the superior one gives superior nasal and superior temporal arteries. The inferior branch gives inferior nasal and inferior temporal arteries. There is also a single venular trunk of central retinal vein (white arrow) divided into superior (S) and inferior (I) branches; the superior one gives superior nasal (SN) and superior temporal (ST) veins. The inferior branch gives inferior nasal (IN) and inferior temporal (IT) veins.

## 3. Discussion

To the best of our literature review, there is not any similar reported case of retinal vein occlusion with 3-quadrant involvement [4–9]. In our patient, three quadrants of involved draining venous branches indicate that obstruction is at the level of lamina cribrosa as a known place for obstruction of central retinal vein [1–2]. Sparing of inferotemporal trunk indicates that superior and inferior trunks join after lamina cribrosa, but engorgement of this branch may show its proximity to site of obstruction [2]. This means that two trunks join in a very short distance after lamina cribrosa.

This kind of variation that spares one quadrant may influence prognosis and also treatment. In this condition, there is one spared quadrant that can help venous drainage. It potentially can cause better response to anti-VEGF therapy and better final prognosis. This hypothesis should be investigated in future comparative studies; however, due to rarity of this variation, it would not be easy to study. Hemicentral retinal vein occlusion is a relatively common presentation [5] in retina clinic, but 3-quadrant involvement has not been reported yet. Khan and Chong [4] reported a case of retinal vein obstruction involving two opposing retinal quadrants in an 88-year-old woman. In their case, inferotemporal and supranasal quadrants were involved as a result of joining venous branches draining these quadrants when passing lamina cribrosa.

One of the differential diagnoses could be optociliary shunt vessels. Optociliary shunt vessels are diagnosed based on fundus examination with their classic appearance as tortuous vascular loops that begin and end on the optic disc [10]. Vascular remodeling cannot make a new separate branch in its whole way with multiple branching and specific draining region. Furthermore, the patient was an alert 50-year-old male without any previous history of visual disturbances (due to an old RVO) in his both eyes.

There are well-known variations in central retinal artery [2], but there are very few reports about variations in central retinal vein [3]. We think lack of enough information regarding the most important venous drainage system of retina needs more notice.

We suggest more population-based studies of retinal vein and artery anatomical variations with fluorescein angiographic evaluation or newer noninvasive methods based on smart phone fundoscopy, optical coherence tomography angiography, or adaptive optics [8–9]. Cho et al. used a noninvasive method by the XyCAM RI (Vasoptic Medical, Inc.) using laser speckle contrast imaging to reveal retinal blood flow and vascular anatomy [8]. These studies can help better understanding of retinal vein and artery occlusion underlying mechanisms and may provoke future surgical or medical treatments.

## 4. Conclusion

In unusual presentations of retinal vascular branch obstructions, considering retinal vascular anatomy variations would help us to explain the clinical presentation more precisely.
